# Multi-state model for studying an intermediate event using time-dependent covariates: application to breast cancer

**DOI:** 10.1186/1471-2288-13-80

**Published:** 2013-06-20

**Authors:** Carolina Meier-Hirmer, Martin Schumacher

**Affiliations:** 1 Infrapôle Paris Saint-Lazare, SNCF, 66, rue Franklin prolongée, Courbevoie 92400, France; 2 Institute of Medical Biometry and Medical Informatics, University Medical Center Freiburg, Stefan-Meier-Straße 26, Freiburg 79104, Germany

## Abstract

**Background:**

The aim of this article is to propose several methods that allow to investigate how and whether the shape of the hazard ratio after an intermediate event depends on the waiting time to occurrence of this event and/or the sojourn time in this state.

**Methods:**

A simple multi-state model, the illness-death model, is used as a framework to investigate the occurrence of this intermediate event. Several approaches are shown and their advantages and disadvantages are discussed. All these approaches are based on Cox regression. As different time-scales are used, these models go beyond Markov models. Different estimation methods for the transition hazards are presented. Additionally, time-varying covariates are included into the model using an approach based on fractional polynomials. The different methods of this article are then applied to a dataset consisting of four studies conducted by the German Breast Cancer Study Group (GBSG). The occurrence of the first isolated locoregional recurrence (ILRR) is studied. The results contribute to the debate on the role of the ILRR with respect to the course of the breast cancer disease and the resulting prognosis.

**Results:**

We have investigated different modelling strategies for the transition hazard after ILRR or in general after an intermediate event. Including time-dependent structures altered the resulting hazard functions considerably and it was shown that this time-dependent structure has to be taken into account in the case of our breast cancer dataset. The results indicate that an early recurrence increases the risk of death. A late ILRR increases the hazard function much less and after the successful removal of the second tumour the risk of death is almost the same as before the recurrence. With respect to distant disease, the appearance of the ILRR only slightly increases the risk of death if the recurrence was treated successfully.

**Conclusions:**

It is important to realize that there are several modelling strategies for the intermediate event and that each of these strategies has restrictions and may lead to different results. Especially in the medical literature considering breast cancer development, the time-dependency is often neglected in the statistical analyses. We show that the time-varying variables cannot be neglected in the case of ILRR and that fractional polynomials are a useful tool for finding the functional form of these time-varying variables.

## Background

In this article the disease course of primary, non-metastatic breast cancer patients is analysed taking first isolated locoregional recurrence (ILRR) of the tumour into account as an intermediate event. In this context, usually separate analyses are carried out for each endpoint and also for the intermediate events. As pointed out by [1] “these separate analyses are not completely satisfying, since they fail to reveal the relations between different types of events”. In this article the illness-death model is used. This is a type of multi-state model nowadays widely used in medical research [2] for describing chronic diseases and intermediate events, particularly in oncology [1,3,4].

The presented approach focuses on the analysis of the hazard rate after the first recurrence (intermediate event) in order to understand the influence of the time of occurrence of this event on the further disease development. As endpoints we consider either “distant metastases or death” or “death”. Different methods for modelling and for estimating the hazard function after recurrence are provided. These methods are very general and can be applied to other problems if the course of the disease to be modelled includes an intermediate event. The methods are applied to a dataset containing data from four studies conducted by the German Breast Cancer Study Group (GBSG) see *e.g.*[5].

In the medical literature, the role of the ILRR with respect to the course of the breast cancer disease and the resulting prognosis is not clearly established. Different hypotheses are under debate: On the one hand, the ILRR could be seen as an independent event, which does not alter prognosis after successful removal, or, on the other hand, the ILRR could be a first sign of disease progression [6-8] and even the cause of distant metastases [9]. The impact of time to recurrence on the death hazard after an ILRR was demonstrated in [10].

The models proposed in this article are more general than Markov models for the following reasons: 

•The transition hazards between different disease states are allowed to depend on previous state occupation times. This is necessary for the analysis of the impact of the time to recurrence on the future disease course.

•After the occurrence of an intermediate event, there are two different time scales. The first one is the time since entry into the study or since the diagnosis of the primary tumour, which is usually the time axis used before the intermediate event. The second possible time scale is the time since the occurrence of the intermediate event, which can also play an important role. When constructing the statistical model for a particular disease, it has to be be taken care of which time scale and which previous state occupation times are important for the further development of the disease.

•As pointed out by [11], it is important to use the information contained in covariates changing over time. This information is a potential predictor that should not be neglected or substituted by time-constant covariates.

Concerning the isolated local recurrence of breast cancer, the dependence of the hazard function after the ILRR on the sojourn time is analysed. This means that the effect of the time since occurrence of the ILRR on the further disease development is examined. As stated above, the models proposed in this article are constructed in order to allow time dependence. In medical literature, this time-depending structure of the ILRR is often neglected. In some models the ILRR is even analysed as a “standard” prognostic factor, *i.e.* like a covariate measured at the time of diagnosis of the primary tumour.

In the next section of this article, the illness-death model is described in detail. Based on a Cox regression model, it is shown how different approaches determine the shape of the transition hazard function. A method based on fractional polynomials is proposed for the analysis of the functional form of the time-varying covariates. In Section Application, the presented methods are used to analyse data from the German Breast Cancer Study Group (GBSG). Additionally to the time-varying covariates, also the effects of “standard” prognostic factors are considered. The results are then analysed and discussed.

## Methods

**Figure 1:**
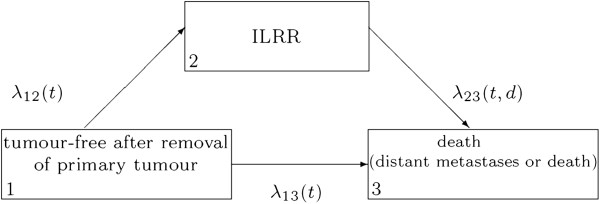
Illness-death model for overall survival (distant disease free survival).

The illness-death or disability model is a very useful tool for describing the course of breast cancer. Figure 1 shows this model in the case of “overall survival”. For the analysis of “distant disease free” survival, the state “death” is replaced by “distant metastases or death”. The variable *t* denotes the time since first diagnosis, *d* denotes the time of ILRR.

When using common regression models for censored data, there are three possible approaches for the estimation of the transition hazards: separate models for every transition (approach **S**), time-dependent covariates (approach **J**) [12], and the stratified model [13]. There are also mixture models, *e.g.* one proposed in [2]. In the following paragraphs, we explain how these methods estimate the transition hazards and which assumptions are (indirectly) made.

The Cox regression model [14] is used throughout the article. The definitions are as follows: *λ*_0_(*t*) denotes the baseline hazard, *λ*_12_(*t*), *λ*_13_(*t*) and *λ*_23_(*t*,*d*) are the hazard functions for the three possible transitions. The parameter vector for the time-dependent covariates is *β*, for the time-invariant covariates *γ* is used. The time-invariant covariates are denoted by *z*. Time-dependent covariates can either depend on *t*, the time since the first diagnosis, or *d*, the time of the intermediate event. We define two time-varying covariates as follows:

$$\begin{array}{lll} {\mathrm{1I}\;}_{\left\{t\geq d\right\}}\; & \cdot d\; & \\ {\mathrm{1I}\;}_{\left\{t\geq d\right\}}\; & \cdot (t-d)\; & \end{array}$$

which we denote only by *d* and *t* - *d* in the following. We are now interested in the functional form with which these variables enter the hazard function. We therefore transform (*t* - *d*) and *d* by a function *f*: *f*(*d*,*t* - *d*). At this stage, the shape of the function *f* is not yet determined. In paragraph Shape of the time-varying covariate some possible choices are proposed for doing this.

Problems can arise when sojourn times and in general random time-dependent covariates are included into multi-state models. For example, cumulative incidence functions cannot longer be estimated in a straightforward way. Such situations were studied by [15] and [11]. The latter paper points out that “studying [...] sojourn times as time-dependent covariates may be useful for testing model assumptions and for investigating their effect on the survival” and treats this kind of problem in section 4.1 [11]. The model mentioned by these authors is equivalent to our straight line model of Section Shape of the time-varying covariate.

The following two paragraphs discuss the joint transition hazards approach **J** and the separate transition hazards approach **S**.

### Joint approach

Approach **J** models transitions 1 →3 and 2 →3 jointly. A time-varying covariate indicates whether the patient is in state 1 or 2 and therefore if the first or the second hazard function is “active”. The model is:

$$\begin{array}{ll} \lambda \;(t)=\lambda_{0}(t)\;\exp\;(\beta^{T}\;{\mathrm{1I}\;\;}_{\left\{t\geq d\right\}}f(d,t-d)+\gamma^{T}\;z), & \end{array}$$

where *1I*_{*t*≥*d*}_ is the time-varying indicator variable and *β* the corresponding parameter. The transition 1 →2 is modelled apart from the other transitions. Therefore the resulting hazard functions are:

(1) $$\begin{array}{c} \;\lambda_{12}(t)=\lambda_{0,12}(t)\;\exp\;(\gamma_{12}^{T}\;z)\\ \;\lambda_{13}(t)=\lambda_{0}(t)\;\exp\;(\gamma^{T}\;z)\\ \lambda_{23}(t,d)=\lambda_{0}(t)\exp\;(\beta^{T}\;f(d,t-d)+\gamma^{T}\;z). \end{array}$$

If this approach is used, the time-invariant covariates *z* are assumed to have the same effect on both transitions to the absorbing state (common parameter *γ*). For transition 1 →2 the effects of the covariates are modelled separately (*γ*_12_ independent of *γ*). The relation between the hazards *λ*_13_ and *λ*_23_ is defined as *c*(*d*,*t* - *d*):= exp (*β*^*T*^*f*(*d*,*t* - *d*)). If only the time-varying indicator is used, *i.e.f*(*d*,*t* - *d*)≡1, the relation *c* = exp (*β*) is constant and the transition hazards 1 →3 and 2 →3 are assumed to be proportional. If *c* = 1, the hazard function is not changed by the intermediate event. If assumption of proportionality is not realistic, time-dependent structures can be added to the function *c* as shown in Table 1.

**Table 1:** **Possible relations between transitions 1 *→ *3 and 2 *→ *3 if approach J is used**

		
J.I	*λ*_23_(*t*,*d*)=*λ*_13_(*t*)	The hazard rate remains the same
J.II	*λ*_23_(*t*,*d*)=*c*·*λ*_13_(*t*)	The hazard rate changes by
		the factor *c* (constant)
J.III.1	*λ*_23_(*t*,*d*)=*c*(*d*)·*λ*_13_(*t*)	*c* depends on *d*, the
		time of ILRR
J.III.2	*λ*_23_(*t*,*d*)=*c*(*t* - *d*)·*λ*_13_(*t*)	*c* depends on *t* - *d*, the
		time since ILRR
J.IV	*λ*_23_(*t*,*d*)=*c*(*d*,*t* - *d*)·*λ*_13_(*t*)	*c* depends on *d* as well as
		on *t* - *d* the time since ILRR

Models J.III (1 and 2) and model J.IV violate the Markov assumption. The estimated parameters of the time-varying covariates allow for testing directly the difference between the hazards before and after the intermediate event. The models are nested and it is therefore possible to use directly a criterion (AIC for example) to assess the goodness of fit. This constitutes an advantage of this approach.

### Separate approach

Approach **S** models all transition hazards separately. The transition *λ*_12_ is modelled like in approach **J** (eq.(1)). Therefore we obtain :

(2) $$\begin{array}{c} \;\lambda_{12}(t)=\lambda_{0,12}(t)\;\exp\;(\gamma_{12}^{T}\;z)\\ \;\lambda_{13}(t)=\lambda_{0,13}(t)\;\exp\;(\gamma_{13}^{T}\;z)\\ \lambda_{23}(t,d)=\lambda_{0,23}(t)\;\exp\;(\beta^{T}f(d,t-d)+\gamma_{23}^{T}\;z). \end{array}$$

The time-invariant covariates are allowed to have different effects on all hazards. The time-dependency of the hazard 2 →3 on the time of recurrence *d* and the time since recurrence *t* - *d* is modelled via the function *f*. Approach **J** is a special case of model **S**.

**Figure 2:**
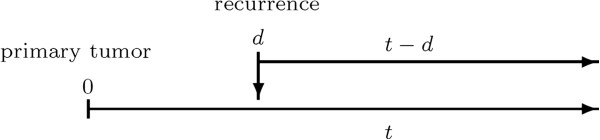
**After the recurrence, two time scales are possible: time since first diagnosis*t* or time since recurrence*t-d*.**

Using this approach, one has to chose the time-scale for the function *λ*_0,23_ what is not self-evident. In equation (2) the time since the first diagnosis *t* is chosen. This approach is often called “clock forward” approach. Figure 2 shows that there is a second possibility: the time since recurrence *t* - *d* can also be used (called the “clock reset” approach). If the time-scale *t* is chosen, only individuals who have already changed to state 2 can be used for the estimation of *λ*_0,23_. It is therefore necessary to account for left-truncation. The implementation of the time-scale *t* - *d* is easier. However, when using time since recurrence, the comparison of the hazard rates before and after recurrence is no longer straightforward.

Approach **S** is a special case of the stratified model [13]. The stratified model assumes different baseline hazards for all transitions. The covariates can be chosen to have identical effects for all transitions or can be stratified by transition whereas the baseline hazards remain different for all three transitions. As the main interest of this article is the modelling of the time-dependent covariates and not the effect of the “standard” covariates, we use approach **S** as described above.

### Shape of the time-varying covariate

Fractional polynomials (FP) are used for the modelling of the function *f*. Fractional polynomials were introduced in [16] and are explained in detail in [17]. In order to carry out model selection using FPs, a sequential selection procedure was proposed in [18]. Our approach is similar to the approach of [19], where time - covariate interaction was investigated using FPs; for a comprehensive overview see [20].

By contrast, in the situation investigated here, fractional polynomials are directly applied on the time-varying covariates *d* and *t* - *d*. The resulting FP gives the function *f* which describes the shape of the impact of the time-dependent covariates on the hazard function *λ*_23_. Using approach **J**, it also determines the relation between function *λ*_13_ and *λ*_23_ as *c*(*d*,*t* - *d*)= exp (*β*^*T*^*f*(*d*,*t* - *d*)).

The procedure is the following: Suppose that *X* is a time-dependent covariate. We are interested in finding the function *f*(.) that describes the impact of the time-dependent covariate on the hazard function. The starting point is a straight line model *f*(*X*) = *β*_1_*X*. In some cases, this is already an adequate description of the relationship, but other models are analysed in order to improve the fit. The extension of the straight line model are power functions *β*_1_*X*^*p*^. The values of *p* are chosen from the set *S* = {-2,-1,-0.5,0,0.5,1,2,3} where *X*^0^ denotes log(*X*). The resulting functions are called one-term fractional polynomials or FP1 functions. The variable that counts the number of FP-terms *m* is set to 1. In this article also FP2 functions are used: $f(X)\;=\;\beta_{1}X^{p_{1}}\;+\;\beta_{2}X^{p_{2}}$, with $p_{1},p_{2}\;\in \;S$. In this case the number of FP-terms increases *m* = 2. If *p*_1_ = *p*_2_, the function $\beta_{1}X^{p_{1}}+\beta_{2}X^{p_{1}}\log(X)$ is used.

In order to find the best model, the 8 different FP1 functions and the 36 different FP2 functions are fitted and compared using the AIC criterion [21].

If there are several time-dependent covariates, linear combinations of the FP of every covariate are used. A model selection procedure treating several covariates is described in [18].

## Application

In this section, the methods described above are applied to a real dataset. In the first paragraph, a description of the dataset is given. Then it is shown how the analysis of the data was carried out.

### Data

Between 1983 and 1989, four studies including patients with primary, histologically proven, non-metastatic breast cancer were carried out by the German Breast Cancer Study Group (GBSG). 2746 patients from 118 clinical institutions entered the studies. The treatment design of the studies is shown in [5], Table 1. The following covariates were registered at primary diagnosis: patient’s age, menopausal status, number of positive axillary lymph nodes, tumour location, tumour size, histologic tumour grade, oestrogen, and progesterone receptor status. Patients were examined at regularly scheduled follow-up visits. The study was carried out with a very small-meshed follow-up scheme in particular in the first years after primary surgery in order to detect any kind of recurrence at the earliest time possible [10]. In the first two years, for example, the examinations took place every 3 month. More details are available in [10]. Follow-up was continued until death. Censoring took place at the end of the study or if follow-up was not possible.

We reduced the data to 2390 patients in order to avoid a missing value problem. This number does not match the number of patients used in [10] as we also excluded patients with survival time equal to zero (*N* = 15). These patients are all censored individuals, lost to follow-up after primary surgery. If ILRR was detected at death we defined the recurrence to have taken place one month earlier. If ILRR and distant metastases were detected at the same time, the time of recurrence was distributed uniformly on an interval of three month before manifestation of the distant disease.

**Figure 3:**
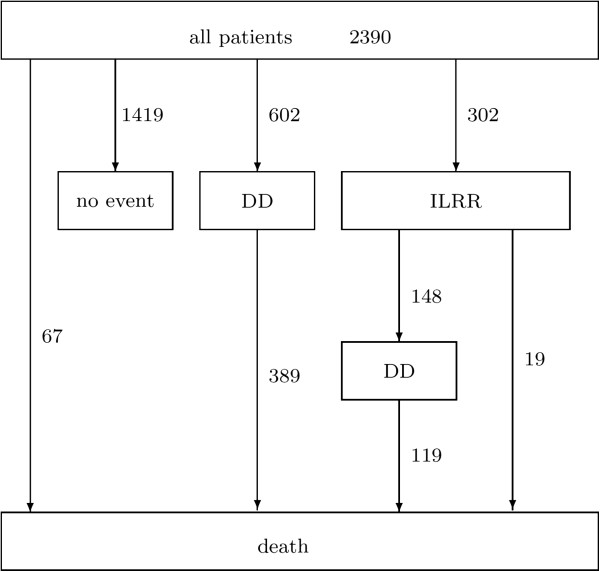
**Observed recurrences and deaths in the four studies.** DD stands for distant disease. ‘No event’ means that these patients are censored without having any of the other events.

Figure 3 shows how many recurrences, distant diseases and deaths were observed in the four studies. 1419 patients had no event. They were still alive without recurrence when the study was terminated. 302 patients experienced an ILRR. After having experienced the ILRR, 148 patients had distant metastases and 19 died without metastases. Among the patients without recurrent event (2088 patients), 602 experienced distant metastases and 67 died.

In order to simplify the application of the proposed methods, the time-invariant covariates were categorized, using pre-defined cutpoints. Only patient’s age, number of positive lymph nodes, tumour size, tumour grade and progesterone receptor status were considered. The other covariates were eliminated within the first model selection using backward elimination at a level *α*=0.01.

### Implementation

Two illness-death models are analysed. In the fist one, the overall survival is estimated, the absorbing state is therefore “death”. Patients with distant disease but without ILRR are censored. In the second model, the endpoint is “distant metastases or death”.

We start with approach **J** which forces the hazard ratio *λ*_23_/*λ*_13_ to have the shape of function *c*. Thereafter, it is investigated if this assumption has to be relaxed by calculating model **S** with *t* as underlying time scale.

Then, the time of recurrence *d* is included into the analysis. Using step-functions to model this time-dependence, it was already shown by [10] that *d* has a linear impact on the overall survival after recurrence. This is why *d* will be included in the model without transformation. It is not known if the effect of time *d* is also linear with respect to distant disease free survival but we will assume this first.

Concerning the time after ILRR, there is a hypothesis that the risk of death is very high just after the recurrence and diminishes with time. But the ILRR could also be an indicator of an accelerated disease development and therefore increase the hazard rate enduringly.

Based on empirical knowledge, a possible shape of the time after recurrence could be exp (-(*t* - *d*)). The more the corresponding parameter estimate is positive, the more serious is the occurrence of an ILRR but the faster is the reduction of the risk to a normal level. In this case, the ILRR could be considered as a new and independent disease that does not alter the hazard rate on the long term. If the parameter estimate is negative, the risk increases up to a saturation level. This corresponds to the hypothesis that the ILRR is a sign of a deterioration of the patient’s health.

The impact of the time after recurrence of the ILRR is first assumed to have the functional form mentioned above, *i.e.* exp (-(*t* - *d*)). However, this assumption has to be checked. In order to confirm this, fractional polynomials are used for the time-varying covariate *t* - *d*. Using approach **J**, it is shown how the shape of the hazard curve changes when time-dependent covariates are ignored. Table 1 gives four possible ways for modelling *c* the relation between hazard 1 →3 and 2 →3. Resuming the assumptions of the previous paragraphs, one obtains:

(3) $$\begin{array}{ll} c(d,t-d)=\exp\;(\beta_{0}+\beta_{1}d+\beta_{2}\exp\;(-(t-d))), & \; \end{array}$$

which is a special case of relation J.IV. By omitting successively *β*_2_, *β*_1_ and *β*_0_, the model is reduced to relation J.III (1 or 2), J.II and J.I, respectively. These models are then compared using the AIC criterion.

In a further step the intuitive choice of the function exp (-(*t* - *d*)) is justified by comparing the resulting hazard function to the hazard function of a time-dependent FP.

The analysis is carried out a second time for the distant disease free survival.

The resulting hazard ratios for approach **J** and **S** are plotted and compared graphically.

The analysis was done in R[22]. The Cox model was estimated using the procedure cph of the rms package written by Frank Harrell [23] and on the coxph procedure of the survival package written by Terry Therneau [24]. It is necessary to use the counting-process notation for survival data in order to account for time-dependent covariates and left-truncation. The modification of the data and the implementation of the programs are described in [13] and [25]. Cumulative baseline hazards were smoothed and first derivatives were calculated by the smoothing function smooth.spline of R. Other methods for deriving the baseline hazard functions like kernel estimators were also applied, but are not shown here. The FP fit was done manually. At present, only software for fitting time-invariant FPs [26] or time - covariate interaction FPs [19,20] exists.

## Results

The following two paragraphs contain the results of the analysis respectively for overall and distant disease free survival.

### Results for overall survival

**Figure 4:**
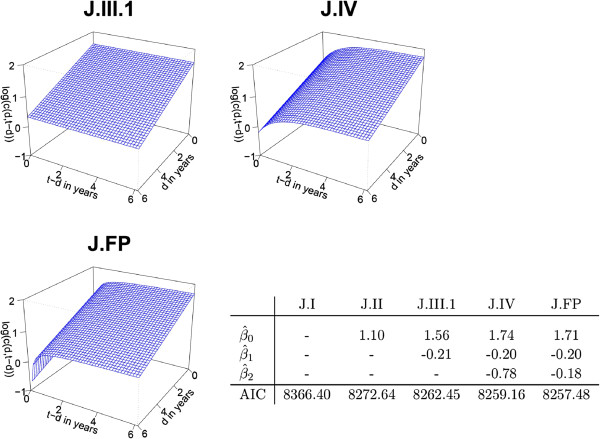
**Outcome variable “death”, approachJ**: Plot of***log (c(d,t-d))* for two of the four models given in Table 1 and the FP approach (J.FP).**The estimates for all models in Table 1 are given. The Akaike information criterion (AIC) of the best J.FP model is 8257.48 and of the model J.I, ignoring the intermediate event, 8366.40. The best FP is (*t* - *d*)^-1^. All coefficients are significantly (*α*-level 0.05) different from zero. With respect to the AIC, the J.FP model is the best one but model J.IV is almost equivalent in terms of the AIC and in terms of the shape of log (*c*(*d*,*t* - *d*)).

The four possible relations between hazard 1 →3 and 2 →3 for approach **J** (*cf.* Table 1), were analysed using equation (3) and “death” as outcome variable. If none of the parameters *β* in equation (3) is included, the resulting model reduces to relation J.I; this model ignores the intermediate event and assumes that the transition hazard 1 →3 is the same as the hazard 2 →3, *i.e.* that the recurrence does not change the hazards. The AIC of this model is 8366.40. If *β*_0_ is included (relation J.II), the hazards are allowed to differ by a constant *c*. In this model, *c* neither depends on *d* (time of recurrence) nor on *t* - *d* (time since recurrence). The AIC of this model is 8272.64. The AIC can be further improved by allowing *c* to depend on *d*, *i.e.* by including additionally *β*_1_. This is a special case of relation J.III.1 (Table 1). The AIC can thus be reduced and is 8262.45 for this model. Figure 4 shows the plots of log (*c*(*t*,*t* - *d*)) for different relations. In comparison to relation J.II (not shown), where log (*c*(*t*,*t* - *d*)) is a horizontal plane, the plane tilts forward using relation J.III.1, indicating that a late recurrence reduces the risk of death. The fourth relation (model J.IV) allows *c* to depend on *d* and on *t* - *d* via exp (-(*t* - *d*)). All parameters contained in equation (3) are estimated. The resulting plot changes from a plane into a curved surface. The parameter estimates of the four models and the corresponding AIC’s are given in Figure 4. The AIC is decreasing between the four models, indicating that model four is the best one of the models proposed (AIC of 8259.16).

In order to know if the function exp (-(*t* - *d*)), based only on expert knowledge and describing the dependence of the hazard function on the time since recurrence (*t* - *d*), accords with reality, FPs were applied on this time-varying covariate. All FP1 and FP2 models were tested and compared using the AIC. The FP1 model (*p*_1_=-1) is the best one and is denoted by J.FP. As can be seen in Figure 4, the J.FP model has a slightly lower AIC but the shape of log (*c*(*t*,*t* - *d*)) remains nearly the same.

**Figure 5:**
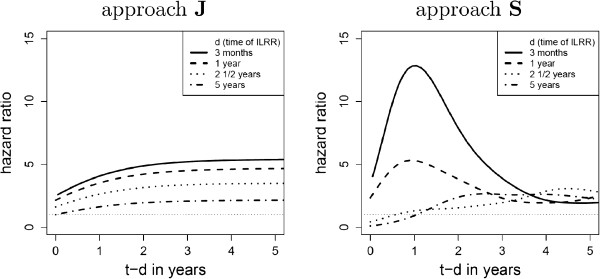
**Outcome variable “death”: Plot of the ratios between the hazard functions before and after the intermediate event for approach JandS**.

In the next step, approach **S** is applied to the data. Also in this case, the model using the expression exp (-(*t* - *d*)) for the influence of the time-dependent covariate *t* - *d* has almost the same quality as the FP model. The first one has an AIC of 1216.56. The FP model uses the transformation *p*_1_=-0.5 for the time-dependent covariate *t* - *d* and has an AIC of 1214.50. In order to analyse whether and how the intermediate event changes the hazard functions, the ratio *λ*_23_(*t*,*d*)/*λ*_13_(*t*) for approach **J** and **S** are compared graphically. Figure 5 shows the hazard ratios for overall survival. The results for approach **J** are derived from the results of model J.IV in Figure 4. The surface is transformed by an exponential function and then cut along the *t* - *d* axis in order to obtain the plot on the left-hand side of Figure 5. The two charts differ importantly from each other. When drawing the hazard ratio *λ*_23_/*λ*_13_ for approach **S**, the complete hazard functions are used including the two baseline hazards *λ*_0,23_ and *λ*_0,13_. For the plot of approach **J**, only the proportional hazard term *c*(*t*,*t* - *d*) is concerned. If the relation between the hazards *λ*_23_ and *λ*_13_ is not proportional or not following the function chosen for *c*(*t*,*t* - *d*), possible variations are forced into the baseline hazard. This is the case in the example shown.

### Results for distant disease free survival

The same analysis was done for the outcome variable “distant metastases or death”. The parameter estimate of the time to recurrence *d* was not significantly different from zero in all approaches (*α*=0.05). This covariate is therefore omitted. Then the functional form of the covariate *t* - *d* is analysed using model J.III.2.

**Figure 6:**
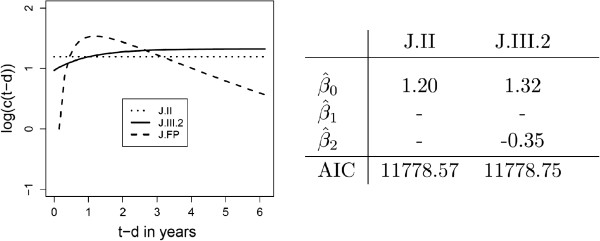
**Outcome variable “distant metastases or death”, approach J**: Plot of***log (c(t-d))* for model J.II, J.III.2 (*cf.*** Table 1) and the FP approach (J.FP). The estimates for model J.II and J.III.2 are given. The AIC of the J.FP model is 11757.02 and 11915.52 for model J.I. The coefficient of exp (-(*t* - *d*)), *i.e.*${\hat{\beta }}_{2}$, is not significantly (*α*-level 0.05) different from zero in model J.III.2. With respect to the AIC, J.FP is the best model.

Figure 6 shows log (*c*(*t* - *d*)) for model J.II, J.III.2 and the FP model (J.FP). With regard to the AIC, model J.II is even better than model J.III.2. The best fitting FP is *m* = 2,*p*_1_ = log,*p*_2_ = log2. In contrast to overall survival, the intuitive functional form exp (-(*t* - *d*)) is not a good choice. J.FP has an AIC of 11757.02 and is therefore better than model J.III.2 which has an AIC of 11778.75. The model J.FP will be retained.

In the next step, approach **S** is applied to the data.

**Figure 7:**
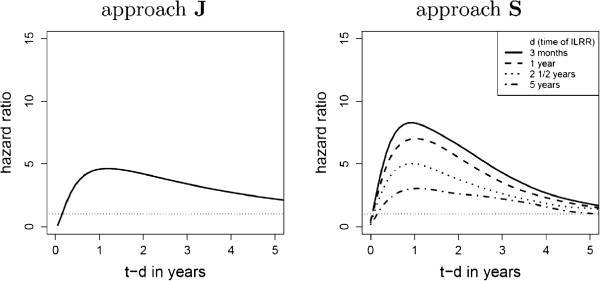
**Outcome variable “distant metastases or death”: Plot of the ratios between the hazard functions before and after the intermediate event for approachJandS**.

Figure 7 shows the hazard ratios for the distant disease free survival time. There is only one curve for approach **J** as the relation *c*(*d*,*t* - *d*) between the hazards does not depend on *d* (the covariate *d* was not significant in the analysis). For approach **S**, the hazard ratio depends indirectly on *d* via the baseline hazards: The baseline hazards themselves do not directly depend on *d* but if the ratio is plotted versus *t* - *d*, the resulting hazards are different for each level of *d*.

The functional form of *t* - *d* was determined by the FP-approach and the FP *m* = 2,*p*_1_ = log,*p*_2_ = log2 was the best model for both approaches, **J** and **S**. The shapes of the hazard ratios are similar for both approaches. The resulting curve of approach **J** corresponds approximately to the curve with *d* = 2.5 years of approach **S**, indicating that approach **J** takes into account a mean value of time of recurrence *d* as this covariate was omitted from the model. Approach **S** shows this dependence on *d* via the baseline hazards.

## Discussion

The often referenced Stanford heart transplant data provided a starting point in illustrating the problem of the comparison of survival curves before and after an intermediate event [27]. Since, disease courses which should be modelled including intermediate events were identified in many medical fields. The course of bone marrow transplantation [28] or the benefit of lung transplantation [29,30] are examples for this development. In the latter two publications, approach **J** was applied including time-varying effects.

In this article, a multi-state model is used for the analysis of breast cancer data including an intermediate event (the ILRR). The study presented here is exactly in the scope of the articles [31] and [32]. These articles deal with illness-death models, where the intermediate event is interval-censored and where there are patients with unknown status at the time of death or follow-up. In our case, ILRR and metastases events are interval-censored as the follow-up took place periodically. The four studies had a median follow-up time between 7 and 9 years, going up to 10 years for the overall survival [10]. The patients are examined at intervals, the ILRR event is the time of the first follow up by which ILRR has occurred. The sojourn time without recurrent event could in this way be overestimated and therefore the lifetime after the recurrent event underestimated. But the follow-up periods are very short in comparison to the study period (*cf.* Section Data). We therefore chose to not take into account the interval-censoring. The bias related to an analysis without interval-censoring should in that case be comparatively small. There was a high number of patients dying from other causes than breast-cancer (67 patients) and autopsy was not compulsory. This could suggest that ILRR or distant disease were present at death but the status was unknown by the study organisers. It has to be taken into account, however, that one third of the patients were older than 60 years at the beginning of the study. So there was a high probability for these patients to die from other causes. An “unknown status” was therefore not incorporated into the analysis that again should not be associated with a large bias.

This article demonstrates that the shape of the hazard ratio in the “illness-death” model depends on the inclusion or exclusion of time depending covariates. Comparisons between model J.I up to model J.IV with “death” as outcome variable confirm that the ILRR should be modelled as a time-dependent intermediate event. The results allow the determination of the impact of the ILRR on the course of the disease. Comparing the results of model J.I and J.II it is obvious that the ILRR is a time-dependent phenomenon and affects the hazard rate. Entering the study, the patients are under risk of ILRR or death. After having experienced an ILRR they are only at risk of death. Therefore a model has to be chosen where the assignment of the observations to different risks is not fixed in advance but dynamic in time. This is ignored in many publications when the patients are grouped retrospectively by recurrence as in [9]. Models that ignore this time-dependency cannot reflect reality and are prone to the so-called time-dependent bias, see *e.g.*[4]. The real effect of the intermediate event cannot be assessed in this way and may be underestimated.

The comparison of model J.II and J.III.1 indicates that the time of recurrence affects overall survival. The later the recurrence appears, the lower is the risk of death. The comparison between model J.III.1 and J.IV implies that the hazard function depends also on time since ILRR. With respect to overall survival (*cf.* Figure 5), approach **J** restricts too much the shape of the hazard ratio. The relation between the hazards in approach **J** does not model the difference between the hazard rates satisfyingly and is therefore not the appropriate model. Approach **S** shows that an early ILRR leads to a sudden increase of the risk of death which decreases relatively fast after the recurrence, but that a late ILRR (after 2 years) only increases the hazard by a factor of about 2.5 which does not longer depend on time since recurrence *t* - *d*. From a medical point of view, it is interesting to know whether the appearance of an ILRR is due to the first disease and expresses the declining health of the patient, or if the ILRR is independent from the first disease. The results show that the ILRR is not completely independent from the first disease. Indeed, the risk of death remains on a higher level after an ILRR (ratio >1). But there is also an independent part: approach **S** indicates that an early recurrence leads to an additional risk, but this risk diminishes after successful removal of the second tumour.

With respect to distant disease free survival, approach **J** models the shape of the ratio correctly, but the dependency on time of recurrence *d* could not be analysed. In fact, in all **J** models this variable was not significant. The model **S** however shows this dependency on *d* via the baseline hazard. This proves that the proportionality assumption between the hazard functions *λ*_13_ and *λ*_23_ in model **J** does not match the data. Whenever the ILRR appears, the risk of distant disease has a peak thereafter. This peak is more pronounced if the ILRR occurs early (Figure 7). After two years, the risk has diminished. In this case, the ILRR can be seen as an independent event. If the recurrence is treated with success, the risk of a distant disease decreases and attains the previous level.

The results also show that fractional polynomials are useful to determinate the functional form of time-varying covariates. Even if the results of the J.IV model using a predefined exponential function are almost equivalent in the case of overall survival, the results show that the use of FPs is the best choice for distant disease free survival.

## Conclusions

Summing up, we have investigated different modelling strategies for the transition hazard after ILRR or in general after an intermediate event. An example is given in which including time-dependent structures alters the resulting hazard function considerably. It is important to realize that there are several modelling strategies and that each of these strategies has certain restrictions and may lead to different results.

It was also shown that fractional polynomials are a useful tool for finding the functional form of the time-varying variables.

Diagnostic methods have changed over the last 20 years. The aim of the paper was to show that the time-varying effect of the ILRR has not to be neglected. The methods developed for doing this are still valid nowadays. The aim of this article was not to reanalyse the four studies of the German Breast-Cancer Study Group.

## Competing interests

We declare that we have no competing interests.

## Authors’ contributions

MS conceived the research topic. CMH explored that idea, performed the statistical analysis and drafted the manuscript. Both authors read and approved the final manuscript.

## Pre-publication history

The pre-publication history for this paper can be accessed here:

http://www.biomedcentral.com/1471-2288/13/80/prepub
